# A CMOS Image Sensor Dark Current Compensation Using In-Pixel Temperature Sensors †

**DOI:** 10.3390/s23229109

**Published:** 2023-11-10

**Authors:** Accel Abarca, Albert Theuwissen

**Affiliations:** 1 EWI Faculty, Electronic Instrumentation Laboratory, Delft University of Technology, Mekelweg 4, 2628 CD Delft, The Netherlands; albert@harvestimaging.com; 2 Now with the International Iberian Nanotechnology Laboratory, Avenida Mestre José Veiga s/n, 4715-330 Braga, Portugal; 3 Harvest Imaging, Witte Torenwal 8E 2.1, 3960 Bree, Belgium

**Keywords:** CMOS image sensor, dark current compensation, in-pixel temperature sensors

## Abstract

This paper presents a novel technique for dark current compensation of a CMOS image sensor (CIS) by using in-pixel temperature sensors (IPTSs) over a temperature range from −40 °C to 90 °C. The IPTS makes use of the 4T pixel as a temperature sensor. Thus, the 4T pixel has a double functionality, either as a pixel or as a temperature sensor. Therefore, the dark current compensation can be carried out locally by generating an artificial dark reference frame from the temperature measurements of the IPTSs and the temperature behavior of the dark current (previously calibrated). The artificial dark current frame is subtracted from the actual images to reduce/cancel the dark signal level of the pictures. In a temperature range from −40 °C to 90 °C, results show that the temperature sensors have an average temperature coefficient (TC) of 1.15 mV/°C with an inaccuracy of ±0.55 °C. Parameters such as conversion gain, gain of the amplifier, and ADC performance have been analyzed over temperature. The dark signal can be compensated in the order of 80% in its median value, and the nonuniformity is reduced in the order of 55%.

## 1. Introduction

Over the last few decades, the semiconductor industry has experienced exponential growth. Semiconductors are widely used in different sensing applications ranging from surveillance to medical devices, as well as environmental applications and beyond [1,2,3,4,5]. One of the most important results of this industry is the CMOS image sensor (CIS) [6,7]. The CIS active pixel sensor (APS) industry has rapidly developed, pushed by the growing market for portable devices. The CMOS APS is widely used because of its high integrability, low cost, and low power consumption [8,9,10]. The dark current is one of the most important performance parameters in the CMOS image sensor (CIS), where a low dark current is preferred. However, keeping low dark current levels is a challenge due to the continuous downscaling of CMOS technology [11,12,13].

In the CIS, one of the major contributors to fixed pattern noise (FPN) corresponds to the dark current [14,15]. A large dark current directly affects the CIS performance in terms of noise, pixel nonuniformity, and reduction in dynamic range [16,17]. The dark current linearly increases with the exposure time and exponentially with the temperature. The dark current doubles every ~5–10 °C [18,19,20,21]. The most common technique for dark current compensation is to take a dark reference frame at a certain exposure time with a closed shutter and subtract this reference frame from the actual images. But, in order to circumvent any dark current variation, the temperature must be maintained constant. Additionally, devices such as mobiles do not have a mechanical shutter to obtain a dark reference frame. In our previous work [21], a novel technique for dark current compensation without the need for constant temperature and mechanical shutter was proposed. In this paper, the dark current compensation technique is extended for different temperatures and exposure times.

The dark current corresponds to an offset signal of the pixel generated under no-light conditions. Although the dark current is not really noticeable under normal illumination and short exposure times, it becomes of relevance when images require long exposure times under low light conditions or with images taken at high-temperature operations. The dark current in pixels using pinned photodiodes is mainly due to the depletion dark current (at low temperatures) and due to the diffusion dark current (at high temperatures) [22,23,24].

Different techniques for dark current compensation have been proposed. In [25], the dark current of hundreds of hot pixels is used as a temperature indicator across the pixel array in a temperature range between −40 °C and 8 °C. The calibrated dark current of the hot pixels predicts the dark current levels of the rest of the pixels. However, no information about the inaccuracy of the hot pixel temperature sensors is provided, and the temperature range is limited between −40 °C and 8 °C. Another technique for dark current compensation was proposed by [26]. In [26], the compensation involves using an active photodiode connected to the inverting input of a Miller differential amplifier, which is compared to a dummy-reference-shielded photodiode connected to the noninverting input of the amplifier. The video signal (active photodiode) and the offset level (dark current from the dummy photodiode) are sampled and subtracted, resulting in a video signal free of dark current. However, the mismatch between the active photodiode and the dummy-shielded photodiode is not addressed in this work.

This paper proposes a technique for dark current compensation, creating an artificial dark reference frame at a certain exposure time by using the temperature information of the pixel array provided by IPTSs. The integration of temperature sensors has been presented in our previous works. It was performed first by using the parasitic substrate bipolar temperature sensor pixel (Tixel) [27,28] and then by using the imaging pixel itself as a temperature sensor [29]. In this paper, the CIS and the IPTSs are characterized in a temperature range between −40 °C and 90 °C.

This paper is organized as follows. Section 2 explains the CIS device with in-pixel temperature sensors. In Section 3, the dark current compensation technique is presented. Measurement results are shown in Section 4. A conclusion is given at the end of this paper.

## 2. CMOS Image Sensor with In-Pixel Temperature Sensors

**Figure 1:**
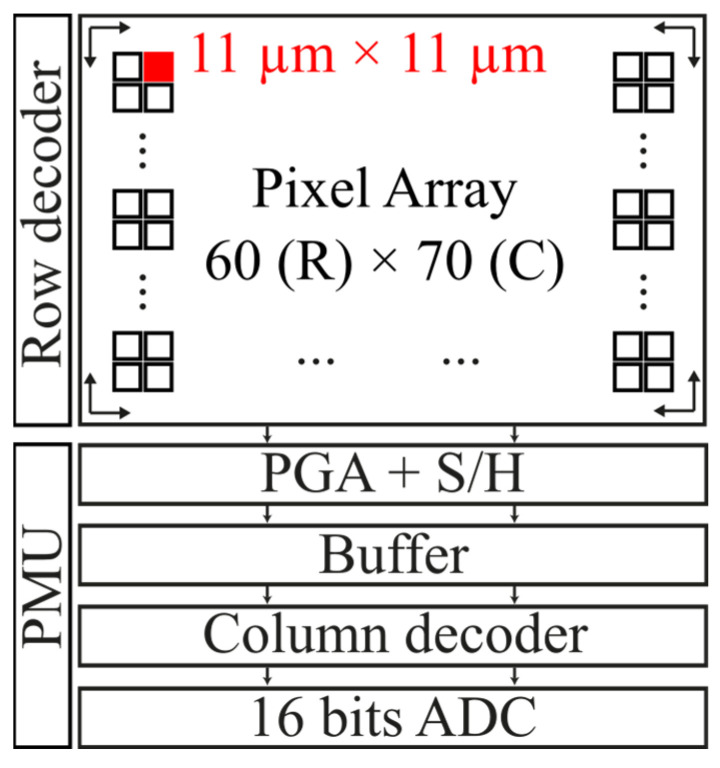
Block diagram of the CMOS image sensor.

The test CIS consists of a 60-row (R) × 70-column (C) pixel array, a programmable gain amplifier (PGA), a sample and hold (S/H), an output buffer, a power management unit (PMU), row and column decoders, and an external 16 bits ADC [30]. Figure 1 shows the CIS block diagram. The PGA provides different gain levels to the video/temperature signal and, together with the S/H, performs the correlated double sampling (CDS) operation (the PGA and S/H are explained in more detail in Section 2.2). The output buffer is used to properly drive the external ADC. Row and column decoders are used to select each pixel position across the pixel array. The PMU generates all on-chip power supplies, such as bandgap references and current sources. The pixel array contains both types of sensor pixels and temperature sensors. Both types of sensors make use of the same read-out system. The size of the 4T pixel is 11 × 11 µm^2^.

### 2.1. In-Pixel Temperature Sensor Based on 4T CMOS Image Sensor

**Figure 2:**
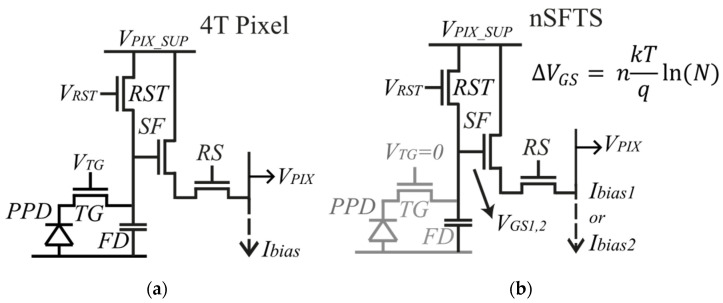
(**a**) 4T pixel. (**b**) nMOS source follower temperature sensor.

The image pixel of the CIS is based on a CMOS 4T (4-transistor) architecture, as shown in Figure 2a. The typical 4T pixel consists of a pinned photodiode (PPD), a floating diffusion (FD), and four transistors: the transfer gate (TG), the reset transistor (RST), the source follower (SF), and the row select (RS). This architecture offers low noise, low dark current (thanks to the PPD), and high quantum efficiency [31].

The nMOS source follower (nSFTS) of the 4T pixel itself is used as a temperature sensor, as shown in Figure 2b. Therefore, the pixel acts either as an image pixel or as a temperature sensor, but not simultaneously. The nSFTS does not incur any additional area. Certain conditions must be fulfilled when the nSFTS is used. The TG transistor is switched off ($V_{TG}=0$) to avoid any light-induced charge that might affect the FD voltage node, as shown in Figure 2b. Also, the gate voltage of the RST transistor ($V_{RST}$) has to be greater than the sum of the pixel supply ($V_{PIX\_SUP}$) and the threshold voltage of RST ($V_{TH\_RST}$); thus, $V_{RST}>V_{PIX\_SUP}+V_{TH\_RST}$. In this way, the gate voltage of the SF transistor equals to $V_{PIX\_SUP}$, and hence, the output voltage ($V_{PIX}$) corresponds to $V_{PIX}=V_{PIX\_SUP}-V_{GS}$, where $V_{GS}$ corresponds to the gate-source voltage of the SF transistor. As $V_{PIX\_SUP}$ is a fixed value, then $V_{PIX}$ depends only on $V_{GS}$ variations. If there is any light-induced variation in the FD (even with TG switched off), this offset can be canceled/reduced with the CDS action. When the SF transistor is biased in the subthreshold region, it exhibits an exponential characteristic depending on temperature [32]:

(1) $$I_{bias}=I_{DS}\cdot e^{\frac{V_{GS}-V_{TH}}{nV_{T}}}\to V_{GS}=nV_{T}\ln\left(\frac{I_{bias}}{I_{DS}}\right)-V_{TH}$$

where $I_{bias}$ is the biasing current (at column level), $I_{DS}$ is the saturation current, $V_{TH}$ is the threshold voltage of the SF transistor, n is a process-dependent factor, and $V_{T}=kT/q$ is the thermal voltage. When the SF is biased by ratiometric currents, the differential gate-source output voltage ($∆V_{GS}$) is proportional to absolute temperature (PTAT) [32,33]:

(2) $$∆V_{GS}=V_{GS2}-V_{GS1}=n\frac{kT}{q}\ln\left(N\right)$$

where $V_{GS2}$ and $V_{GS1}$ correspond to the gate-source voltage when the SF transistor is biased at currents $I_{bias2}$ and $I_{bias1}$, respectively, and $N=I_{bias2}/I_{bias1}$. Thus, a temperature measurement is obtained from $∆V_{GS}$.

**Figure 3:**
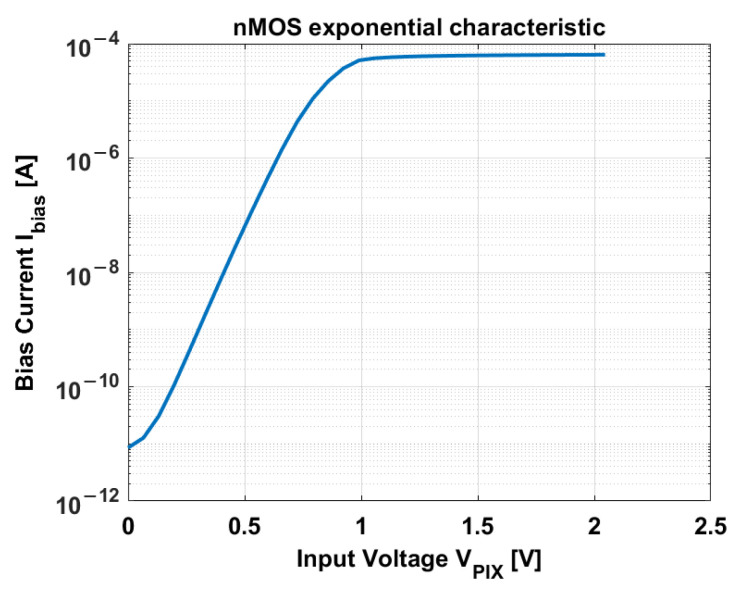
I-V characteristic of the source follower transistor [33].

The in-pixel source follower occupies a minimum area of 0.5 × 0.8 µm^2^. A minimum size nMOS transistor is used to maximize the fill factor of the pixel. The biasing currents are chosen accordingly to bias the SF in the exponential region. A simulation of the I–V characteristic of the source follower is shown in Figure 3.

**Figure 4:**
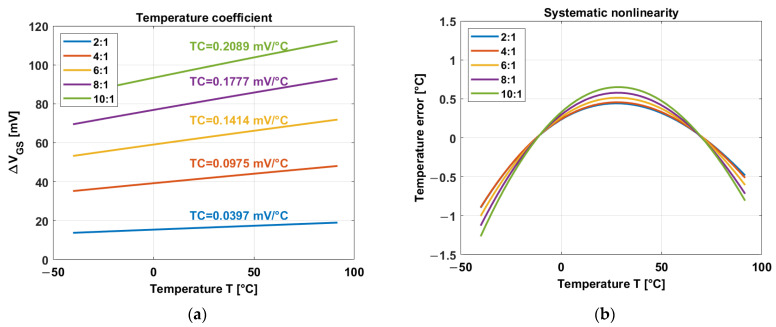
(**a**) Temperature coefficient of the nSFTS for different current ratios [33]. (**b**) Systematic nonlinearity of the nSFTS after a 1^st^-order curve fitting [33].

The exponential characteristic extends from 100 pA to 10 µA. A 1 µA current is chosen as the unit biasing current. Simulations of different current ratios are performed, as shown in Figure 4a.

Figure 4a clearly shows that a higher temperature coefficient is proportional to the current ratio N. It also shows higher output $∆V_{GS}$. Higher $∆V_{GS}$ is desirable (as well as higher TC) in order to cover the dynamic range of the read-out circuit.

**Figure 5:**
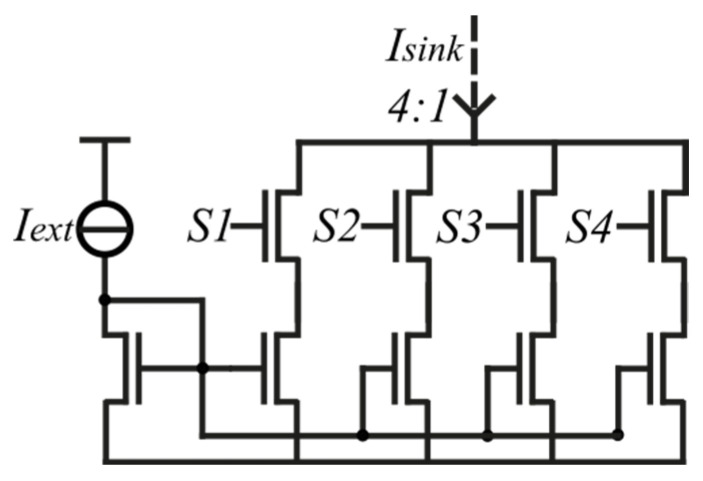
Circuit of the current sink generator. The circuit is supplied by an external current ($I_{ext}=1\;\mu A$). Depending on the switches (on/off), the current sink generates a specific current ratio (in this case, 4:1).

However, linearity becomes worse with higher current ratios, as shown in Figure 4b. The systematic nonlinearity temperature error ($e_{T}$) is calculated by the difference between $∆V_{GS}$ and the linear fit ($V_{fit}$) divided by the temperature coefficient (TC): $e_{T}=\left(∆V_{GS}-V_{fit}\right)/TC$. For instance, current ratio N = 2 exhibits good nonlinearity but small TC and $∆V_{GS}$. A fair compromise between a relatively high TC (and output $∆V_{GS}$) and good linearity is reached with N = 4. The ratio 4:1 is generated by a regular sink current mirror, as shown in Figure 5. Switches S1, S2, S3, and S4 are turned on and off in order to generate a certain current ratio, such as 4:1.

### 2.2. Read-Out System

**Figure 6:**
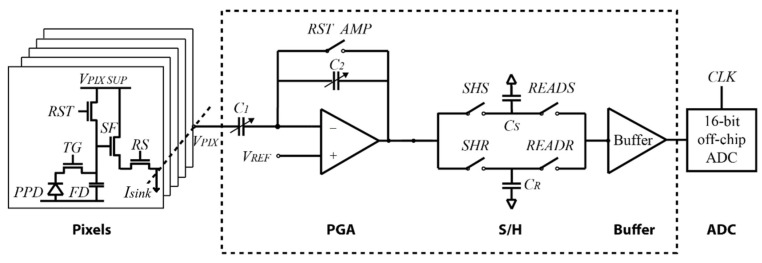
Block diagram of the read-out circuit [33].

The on-chip read-out system consists of column PGAs, the S/H, and the output buffer. Gains 1×, 2×, 4×, 8×, and 16× are provided by the PGA depending on the ratio of the input and feedback capacitors $C_{1}$ and $C_{2}$, respectively. The gain corresponds to $G=C_{1}/C_{2}$. Sixteen capacitors of 100 fF in parallel correspond to the $C_{1}$ bank of capacitors, and $C_{2}$ contains four capacitors of 100 fF in parallel; for example, select all the capacitors in $C_{1}$ and only one in $C_{2}$, then G=16. A decoder selects the gain. The S/H circuit is composed of $C_{S}$ (signal capacitor) and $C_{R}$ (reference capacitor) corresponding to the analog memories. The S/H circuit is controlled by signals *SHS* and *SHR* for the sample phase and by signals *READS* and *READR* for the read-out phase (off-chip ADC). A detailed block diagram of the read-out system is shown in Figure 6.

The column amplifier realizes the CDS operation, which is a well-known technique to cancel the kTC noise of the reset action at pixel level as well as the offset of the amplifier. As the CDS operation samples two signals in two different phases, it becomes natural to use the same technique to sample $V_{GS1,2}$ of the nSFTSs.

The read-out of the nSFTSs works as follows. When the nSFTS is biased at 1 µA, the output voltage $V_{GS1}$ is stored in $C_{1}$, and the offset plus the reference voltage ($V_{OS}+V_{REF}$) are sampled and stored in the analog memory $C_{R}$. In the next phase, the output signal $V_{GS2}$ (biased at 4 µA) is stored in $C_{1}$ as well, obtaining the differential gate-source voltage ($\Delta V_{GS}=V_{GS2}-V_{GS1}$), which is amplified by the gain $G=C_{1}/C_{2}$ and stored with $V_{OS}+V_{REF}$ in $C_{S}$. In such a manner, the stored values in both analog memories are subtracted canceling the offset: $V_{out}=\left(G\cdot \Delta V_{GS}+V_{REF}+V_{OS}\right)-\left(V_{REF}+V_{OS}\right)=G\cdot \Delta V_{GS}$.

## 3. Dark Current Compensation Technique

**Figure 7:**
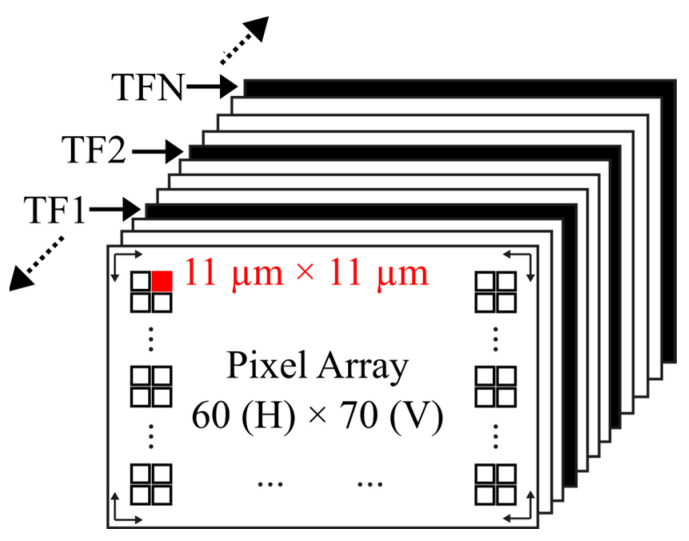
Temperature frames taken in between image frames.

The proposed dark current compensation technique is based on the use of the CMOS imager pixel as a temperature sensor. The idea is to take a temperature frame (TF) in between the video/image frames, as shown in Figure 7 [21].

The TF provides the absolute temperature (from $\Delta V_{GS}$) per pixel. In consequence, temperature information, as well as thermal distribution, is locally obtained. The differential voltage $\Delta V_{GS}$ is PTAT, as shown in Equation (3):

(3) $$∆V_{GS}=a\cdot T+b$$

where a and b are constants.

It is known that the dark current follows the Arrhenius law, as do many thermally activated processes; thus, $I_{dark}=I_{0}\cdot exp\left(-∆E/kT\right)$, where $I_{0}$ corresponds to the dark current and ∆E is the activation energy, both factors depend on temperature. The Meyer-Neldel rule (MNR) is an empirical law that is frequently observed in processes following the Arrhenius law [20]. The MNR states that a logarithm of the factors has a linear dependence on the activation energy. Therefore, rearranging the dark current over temperature in the form of:

(4) $$I_{dark}=c\cdot e^{d\cdot T}$$

factors c and d can be treated as constants [20].

By combining Equations (3) and (4), then $I_{dark}$ depends on $∆V_{GS}$:

(5) $$I_{dark}=c\cdot e^{d\cdot \left(\frac{∆V_{GS}-b}{a}\right)}$$

Equation (6) exhibits the linear dependence of the dark signal ($S_{dark}$) on the exposure time ($t_{exp}$).

(6) $$S_{dark}=I_{dark}\cdot t_{exp}$$

Replacing Equation (5) in Equation (6), the absolute dark signal is obtained from the dark current value (through $∆V_{GS}$) at a certain exposure time, as shown in Equation (7):

(7) $$S_{dark}=c\cdot e^{d\cdot \left(\frac{∆V_{GS}-b}{a}\right)}\cdot t_{exp}$$

By using this algorithm, it is possible to obtain the absolute dark signal of each pixel across the pixel array, and an artificial dark reference frame can be generated from the temperature information. Therefore, the absolute temperature information provided by the nSFTS can be utilized to generate an artificial dark frame without the need for a mechanical shutter and the request for constant temperature. Constants a, b, c, and d, are calculated from measurements.

## 4. Measurement Results and Discussion

**Figure 8:**
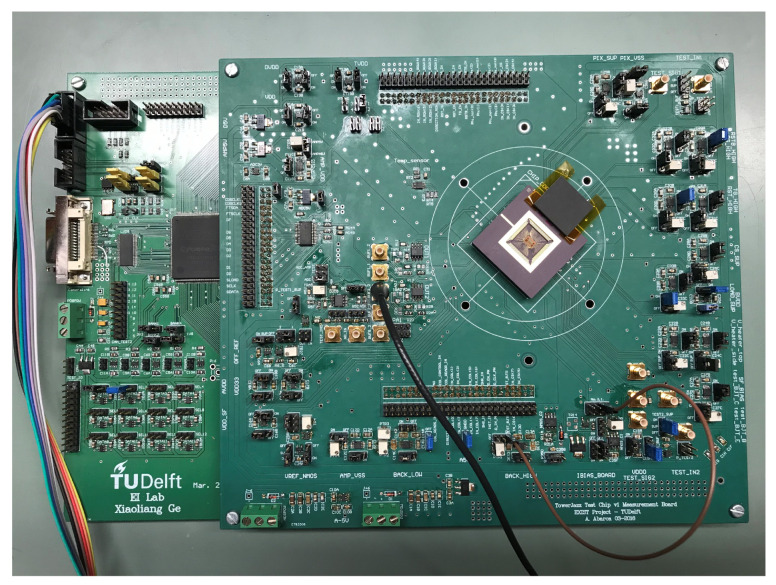
PCB of the CIS device under test.

The measurement setup is composed of a PCB with the power regulators and the ADC, an FPGA producing all control signals (of the CIS and ADC), a PC with Quartus, LabView and Matlab for processing, and a temperature-controlled oven VT7004 with a reference calibrated Pt-100 temperature sensor. Measurements were performed over a temperature range between −40 °C and 90 °C in steps of 10 °C. Figure 8 shows the PCB of the CIS device.

The CIS device was fabricated with a standard CIS 0.18 µm TowerJazz technology.

**Figure 9:**
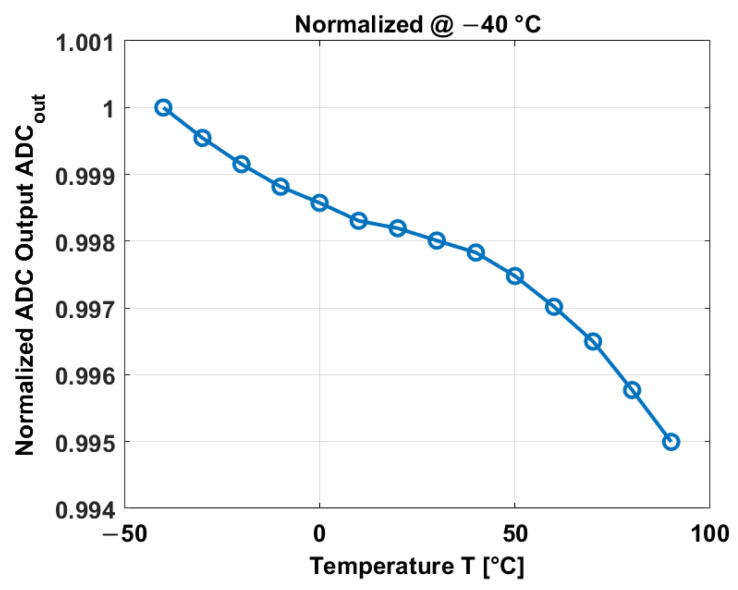
Normalized ADC output.

The PCBs have been measured over temperature without including the CIS device in order to investigate the influence of the discrete components on the CIS device. A constant input of 50 mV has been applied to the input of the ADC, and the ADC output is shown in Figure 9.

The ADC output exhibits small changes over temperature. It only changes 0.5% in a temperature range between −40 °C and 90 °C. Thus, the influence of the PCB-ADC can be neglected.

### 4.1. Parameters of the Pixel

The dark current is commonly expressed in electrons ($e^{-}$), but the $I_{dark}$ is obtained in digital number (DN) from the measurements. The conversion gain (CG) is used to convert DN into $e^{-}$. Another parameter involved in obtaining the dark current is the gain of the PGA (which is used to cover the full dynamic range of the read-out). The dark current is obtained by using a gain of 2. Equation (8) shows the calculation of the $I_{dark}$ in $e^{-}$ by using these two parameters.

(8) $$I_{dark}(e^{-})=\frac{I_{dark}(DN)}{LSB\cdot CG\cdot G}$$

where $I_{dark}(DN)$ corresponds to the dark current in *DN*, $I_{dark}(e^{-})$ is the dark current expressed in $e^{-}$, and LSB corresponds to the least significant bit of the ADC, which is 30 µV.

**Figure 10:**
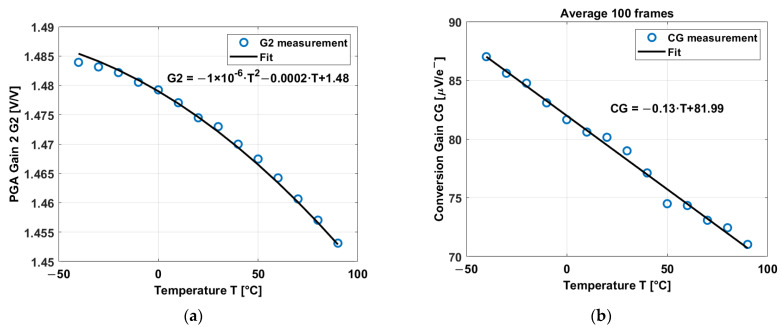
(**a**) PGA Gain 2 over temperature. (**b**) Conversion gain of the CIS device over temperature.

Measurements of the G and CG over temperature are shown in Figure 10.

The gain of the PGA decreases over temperature at a rate of 0.0002 V/V/°C. It exhibits a curvature of 1 × 10^−4^%. Also, the gain is not exactly 2 but 1.475 V/V at room temperature, and most probably it is affected by parasitics. The conversion gain has a temperature coefficient of −0.13 µV/$e^{-}$/°C. This behavior has been reported in [34,35], but in this paper, the temperature range has been extended compared to [34,35].

### 4.2. Dark Signal and Dark Current

**Figure 11:**
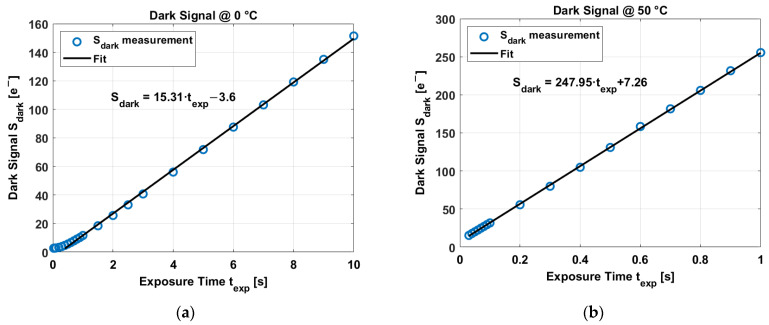
(**a**) Dark signal over exposure time at 0 °C. (**b**) Dark current over exposure time at 50 °C.

After averaging 100 frames and all pixels, the dark signal exhibits a linear behavior over the exposure time. In this case, two curves at 0 °C and 50 °C are shown in Figure 11.

The dark current corresponds to the slope of the dark signal over exposure time, at 0 °C corresponds to 15.31 $e^{-}/s$, and at 50 °C is 247.95 $e^{-}/s$. The dark signal in Figure 11a exhibits a “dark signal floor” of ~2.8 $e^{-}$ at low exposure times. This corresponds to the “noise floor” limited by the read-out electronics.

**Figure 12:**
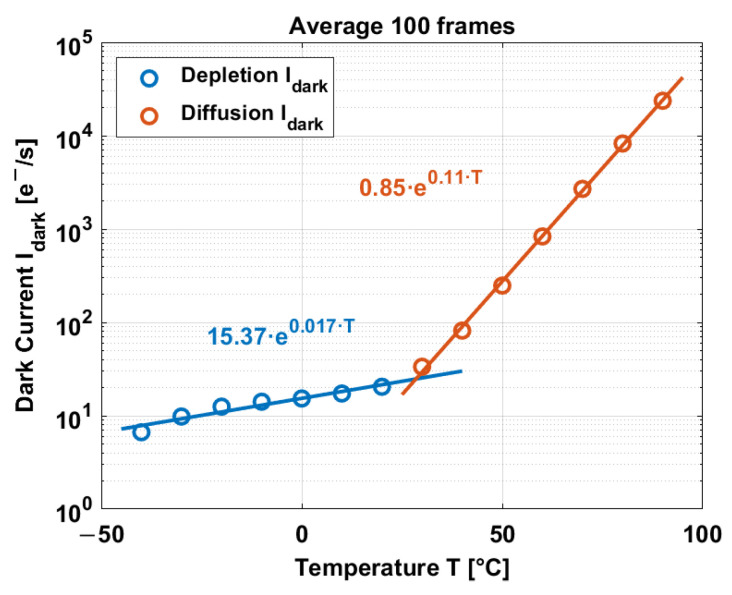
Dark current over temperature.

In consequence, the dark current is obtained at different temperatures and is displayed in Figure 12.

The dark current exhibits an exponential behavior over temperature, where the depletion dark current dominates at low temperatures, while the diffusion dark current dominates at high temperatures. The dark current at low temperature (below 30 °C) and at high temperature is fit by an exponential curve giving the relation between $I_{dark}$ and T. Factors c and d (from Section 3): c = 15.37 e^−^/s, d = 0.017 °C^−1^ for depletion $I_{dark}$; c = 0.85 e^−^/s, d = 0.11 °C^−1^ for diffusion $I_{dark}$.

### 4.3. Temperature Sensor

**Figure 13:**
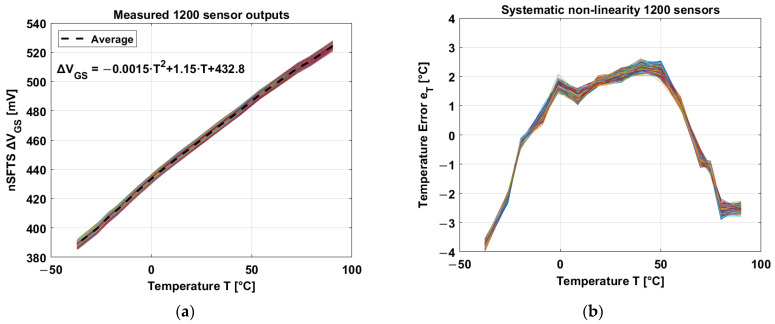
(**a**) Differential gate-source voltage over temperature, the TC is 1.15 mV/°C [21]. (**b**) Systematic nonlinearity.

The nSFTSs have good linearity and accuracy in a temperature range between −40 °C and 90 °C. After averaging 1200 temperature sensors, the average temperature coefficient corresponds to 1.15 mV/°C, as shown in Figure 13a. Constants a and b (from Section 3) correspond to (the curvature of $∆V_{GS}$ is negligible) a = 1.15 mV/°C and b = 432.8 mV.

The nonlinearity of the temperature sensors is in the order of ±4 °C after applying a 1^st^-order curve fitting (Figure 13b).

**Figure 14:**
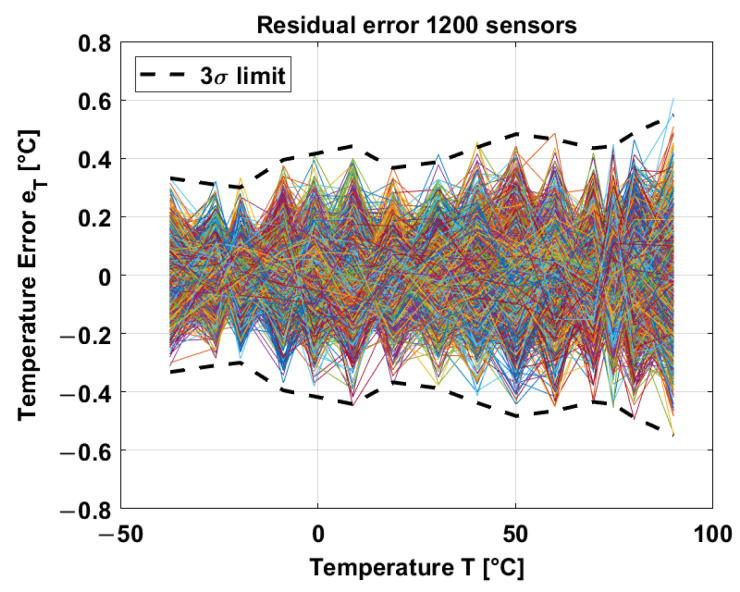
3σ inaccuracy of the in-pixel temperature sensors [21].

After a systematic nonlinearity removal and applying a 2nd-order polynomial, the inaccuracy corresponds to ±0.55 °C, as shown in Figure 14.

### 4.4. Dark Current Compensation Technique

**Figure 15:**
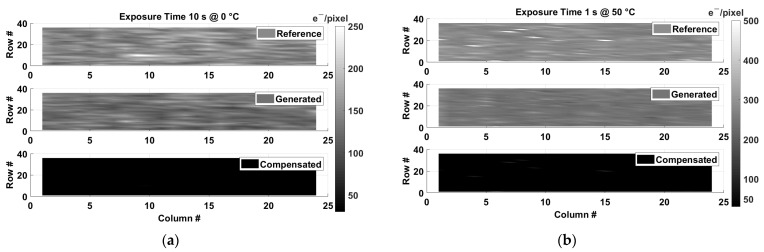
Top: prerecorded dark reference frame; Middle: artificial dark frame; Bottom: subtraction between the dark reference frame and the generated dark frame. (**a**) At 0 °C and at 10 s. (**b**) At 50 °C and 1 s.

An artificial dark reference frame can be generated from the temperature information provided by each nSFTS and the average dark current at a certain exposure time (following the algorithm presented in Section 3). Two artificial dark frames have been generated: one at 0 °C and at 10 s exposure time and another at 50 °C and at 1 s exposure time. These artificial frames are compared to two prerecorded dark reference frames taken with an external closed mechanical shutter on the test CIS device. Figure 15 shows the comparison between the dark reference frames with the closed shutter and the artificial dark frames.

In order to compare the quality of the generated frames, the prerecorded dark reference frames and the artificial frames are subtracted. At 0 °C and 10 s (Figure 15a), the dark signal level (median) reduces from 146 $e^{-}/pixel$ to 25 $e^{-}/pixel$ and the nonuniformity (σ) from 19 $e^{-}$ to 10 $e^{-}$. In the case of 50 °C and 1 s (Figure 15b), the dark signal level (median) reduces from 300 $e^{-}/pixel$ to 50 $e^{-}pixel$, and the nonuniformity (σ) decreases from 40 $e^{-}$ to 15 $e^{-}$.

From Figure 15, it can be observed that the artificial dark frames (middle) are darker than the prerecorded dark frames (top). One of the reasons for this difference is due to the residual offset of the read-out system. Most of the offset is reduced/canceled by the use of correlated double sampling, but still, a residual offset remains, affecting the dynamic range of the temperature measurements. Also, the average dark current is utilized for the dark current compensation instead of the local dark current per pixel. Using the local dark current per pixel might create a more accurate artificial dark frame. Another reason is that there is a difference between the exponential fit of the dark current and the dark current values from measurements (Figure 12). This difference can be up to 6%. Also, the inaccuracy of the temperature sensors contributes to the difference between the artificial frames and the prerecorded dark frames.

**Figure 16:**
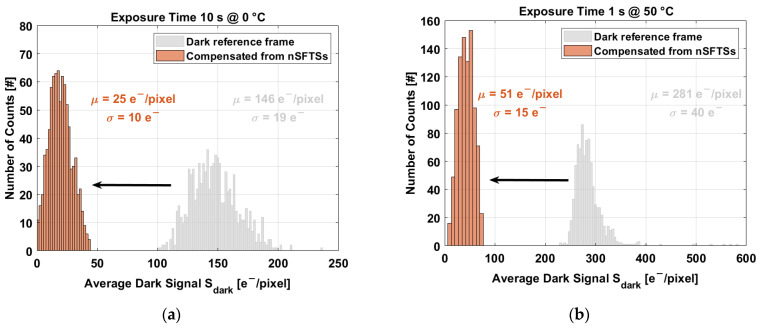
Compensation of the prerecorded dark reference frame with close mechanical shutter. (**a**) At 0 °C and 10 s. (**b**) At 50 °C and 1 s.

The achieved level of compensation of median value and nonuniformity can be clearly observed at the histogram level. Figure 16 exhibits the compensation of the prerecorded dark reference frame at 0 °C (10 s exposure time) and at 50 °C (1 s exposure time).

At 10 s exposure time and 0 °C, the artificial dark frame compensates the prerecorded frame in its median value by 84% and the nonuniformity by 47%, as shown in Figure 16a. Figure 16b exhibits the compensation of the dark frame at 10 s $t_{exp}$ and 50 °C; the compensation of the median value is 82%, and the nonuniformity is 63%.

In terms of the median value, the compensation achieves similar values above 80%, but there is a difference in terms of the nonuniformity. Intuitively, it is expected to have less nonuniformity and a decrease in temperature. In fact, the accuracy of the nSFTSs becomes slightly better at low temperatures, as shown in Figure 14. However, the exponential fit of the dark current at temperatures below 30 °C is not as good as at high temperatures. At low temperatures, the fit $R^{2}=0.90$, while at high temperatures, the fit $R^{2}=0.9996$. The fit $R^{2}$ of the dark current and the accuracy of the temperature sensors explain the level of compensation at low temperatures.

Table 1 shows levels of dark signal compensation for different temperatures and different exposure times. The median value of the dark signal is compensated almost constantly for different temperatures and exposure times, but the nonuniformity increases its compensation over temperature. This behavior is probably due to the fitting accuracy explained in the previous paragraph.

### 4.5. Discussion

This technique has successfully demonstrated the dark current reduction over a temperature range between −40 °C and 90 °C. The 4T pixel itself can be effectively used as a temperature sensor without the need for an extra in-pixel circuit, which, for instance, would reduce the fill factor of the pixel. Also, the dual sensing of the pixel benefits from the correlated double sampling circuit/technique, which is normally utilized in CIS read-outs. Thus, there is no need for a different read-out circuit when the pixel acts as a temperature sensor. Therefore, a full CMOS solution for dark current compensation is achieved. For instance, it is not necessary to use complex implants in the active area of the pixel to reduce dark current, as in [36]. Furthermore, this technique can be used over a large temperature range in comparison to [25], and there is no need for extra circuitry as in [26].

When the 4T pixel is used as a temperature sensor, the TG is switched off in order to avoid any light-induced affecting the FD. However, the FD itself is sensitive to light. The gate of the SF transistor is tied to the pixel supply, as stated in Section 2.1. If there is any light-induced variation in the FD, this variation will add an offset to $V_{PIX},$ which can be canceled/reduced by the CDS action. In addition, the “exposure time” for temperature measurements is in the order of tens of milliseconds. Thus, the FD might not be affected by this short time. It is worth mentioning that the exposure/integration times for the video signal and temperature signal are not necessarily the same.

## 5. Conclusions

In this work, a novel technique for dark current compensation without the need for a mechanical shutter and constant temperature has been presented. This technique makes use of the temperature dependence of the dark current and the temperature information provided by in-pixel temperature sensors in order to create an artificial dark frame at a certain exposure time. This technique can be applied at any temperature, as well as at any exposure time. The IPTSs are based on the source follower of the 4T pixel itself. Thus, it requires no additional area. The temperature and the nonuniformity of the pixel array are obtained with the PTAT differential gate-source voltage of the source follower when the nSFTS is biased by ratiometric currents. The conversion gain exhibits a decrease of −0.13 µV/$e^{-}$/°C, and the gain of the PGA decreases at a rate of 0.0002 V/V/°C. The nSFTSs exhibit a TC of 1.15 mV/°C and an inaccuracy of ±0.55 °C. In order to prove the efficacy of this technique, two artificial dark frames have been compared to prerecorded dark reference frames. One frame at 0 °C and 10 s exposure time, and another frame at 50 °C and 1 s exposure time. At 0 °C ($t_{exp}$ = 10 s), compensation achieves a level of 84% in its median value and 47% in its nonuniformity, while at 50 °C ($t_{exp}$ = 1 s), the median is compensated by 82% and the nonuniformity by 63%. Dark signal compensations for different temperatures and different exposure times exhibit a reduction in the median in the order of 80%, while the nonuniformity decreases by 50% on average.

## Figures and Tables

**Table 1:** Dark signal compensation at different temperatures and different exposure times.

Temperature [°C]	Exposure Time [s]	Median Value [%]	Nonuniformity [%]
−30	8	84	45
−30	10	83	45
−10	8	83	46
20	2	82	55
20	3	82	53
70	0.5	80	64
90	0.1	80	65

## Data Availability

Data are contained within the article.
